# Directional wave buoy data measured near Campbell Island, New Zealand

**DOI:** 10.1038/s41597-021-01025-3

**Published:** 2021-09-15

**Authors:** Peter McComb, Sally Garrett, Tom Durrant, Jorge Perez

**Affiliations:** 1 Oceanum Ltd, 9a Bow Street, Raglan, 3225 New Zealand; 2 Defence Technology Agency, Jim Titchener Parade, Devonport, 0624 New Zealand; 3 Meteorological Service of New Zealand, 5 Wainui Road, Raglan, 3225 New Zealand

**Keywords:** Physical oceanography, Ocean sciences

## Abstract

The New Zealand Defence Force (NZDF) has established a permanent wave observation station near Campbell Island, south of New Zealand (52 45.71 S, 169 02.54E). The site was chosen for logistical convenience and its unique location adjacent to the highly energetic Southern Ocean; allowing instrumentation typically deployed on the continental shelf to be used in this rarely observed southern environment. From February 2017, a Triaxys Directional Wave Buoy was moored in 147 m depth, some 17 km to the south of the island, with satellite telemetry of the 2D wave spectra at 3-hourly intervals. To date there have been three deployments on locations, yielding some 784 days of data. Validation of the measured significant wave height against co-located satellite altimeter observations suggests that the predominant wave directions are not attenuated by the island. The data provide a valuable record of the detailed wave spectral characteristics from one of the least-sampled parts of the Global Ocean.

## Abstract (summary)

**Table:** 

Measurement(s)	sea state
Technology Type(s)	wave sensing buoy
Factor Type(s)	temporal interval
Sample Characteristic - Environment	ocean
Sample Characteristic - Location	Campbell Island

Machine-accessible metadata file describing the reported data: 10.6084/m9.figshare.15388902

## Background & Summary

The energetic nature of the ocean to the south of New Zealand is well known to mariners. An almost unlimited circumpolar fetch combined with persistent strong winds, creates a climate with frequent storms that occur throughout the year^1^. The swell waves generated in this southern basin propagate throughout the Indian and Pacific Oceans^2^ and make a significant contribution to the wave climate of the northern hemisphere as well^3^. Despite occupying almost a quarter of the world’s sea surface and having high importance to the global wave climate, and the planetary ocean-atmosphere gas fluxes, the Southern Ocean is still the least studied of all the worlds’ ocean areas.

There are good reasons why few *in situ* wave measurements exist for the Southern Ocean. Aside from the rough conditions, the distances from land are vast and the water is deep, which makes measurement campaigns very expensive. With the advent of satellite remote sensing, altimeter data now provides reasonably high spatial and temporal coverage for estimates of the non-directional wave height^4^. However, such data do not provide the precise spectral information that can be obtained from reference wave measuring buoy.

Until recently, the most relevant campaign with spectral observations was the Southern Ocean Flux Station (SOFS); a deep-water mooring located some 500 km south-west of Tasmania for a 24-month period (spread over three deployments from 2012–2015). A Triaxys motion response unit was fitted to the moorings’ surface buoy to allow wave observations to be included in the experiment. The data are reported by Rapizo *et al*.^5^ and were at the time the southernmost spectral dataset in publication (i.e. latitude 47 S).

The New Zealand Defence Force (NZDF) has recognised the absence of detailed wave spectral information for the extensive Southern Ocean areas where the country has marine search and rescue obligations, as well as sovereign and operational patrol responsibilities. Indeed, the current ship class rules for this area specify a design wave case based on northern hemisphere spectra that is transposed onto the southern hemisphere conditions. The consequence of designing and certifying naval ships based on an unvalidated spectral shape could be severe, which has warranted the involvement of the NZDF in a targeted wave data collection program. Aside from critical ship design information, acquisition of detailed spectral data was seen as having benefit in fundamental research of the wave generation and dissipation processes in the Southern Ocean^6^ as well as facilitating general improvements in numerical wave modelling for operational hindcasting and forecasting. These latter benefits are directly addressed in separate programme^7^.

**Fig. 1:**
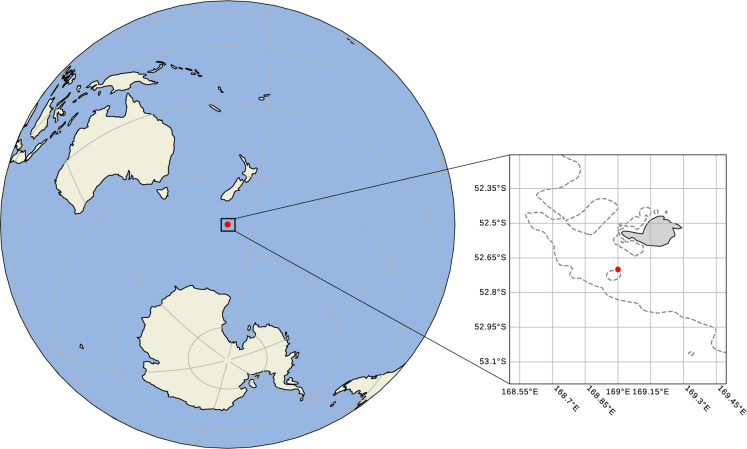
Map showing the location of Campbell Island and the NZDF permanent wave observation site at 52 45.71 S, 169 02.54E, located 17 km south of the island. The 200 m isobath is shown on the inset map, with the buoy location identified by a red dot.

Accordingly, on 8 February 2017 an exploratory observational program was initiated at one of the few exposed locations in the Southern Ocean with continental shelf - allowing a highly responsive spherical instrument to be used. The HMNZS OTAGO deployed a Triaxys Directional Wave Buoy near Campbell Island, which is approximately 600 km south of New Zealand (Fig. 1). The buoy coordinates were latitude 52° 45.71′ S, longitude 169° 02.54′ E and the local depth was 147 m. Since deployment, the buoy reliably transmitted spectral data at 3-hourly intervals with 93% transmission success rate, including a storm event with a maximum individual wave height of 19.4 m^8^. However, on July 27 2017 after 172 days on location, the buoy broke its mooring line and drifted eastwards and was not recovered.

Analysis of the measurements from this initial deployment led to the decision to establish ongoing wave observations at this location. On 2 March 2018, the HMNZS WELLINGTON deployed a replacement Triaxys Directional Wave Buoy, with a revised mooring design to improve the fatigue resistance in these highly energetic waters. This buoy provided data until 19 June 2019; some 474 days with a transmission rate of 91%. During this time, a maximum individual wave height of 23.8 m was measured during May 2018. A third deployment operation was conducted from the HMNZS CANTERBURY on 27 November 2019.

## Methods

Triaxys Directional Wave Buoys are being used for the programme and the standard onboard processing regime used by the manufacturer was adopted. The buoy samples raw data at 4 Hz over 20-minute bursts at 3-hourly intervals, and the onboard spectral processing applies the Maximum Entropy Method to resolve 65 frequency increments (range 0.05 to 0.38 Hz), with directional resolution of 3 degrees (i.e., 121 directional increments). Further information on the Triaxys data processing methodology and instrument validations may be found in the manufacturers’ technical library^9^.

A manual inspection of the resultant spectral estimates was undertaken before upload to the data portals for dissemination. Note that raw and processed spectral estimates are stored on-board for manual download at the annual servicing, while the 1D and 2D spectral files are transmitted by Iridium telemetry in near real-time.

For the first deployment (08/02/2017–27/07/2017) a factory-supplied 15 m rubber compliant section was attached to the buoy, with 185 m of 12 mm Dyneema rope (and midwater buoyancy units) used to anchor the buoy to 600 kg of 32 mm stud-link chain. The mooring revision for the second (02/03/2018–19/06/2019) and third (25/11/2019–25/04/2020) deployments deleted the compliant section as this was the likely failure point under repeated fatigue. Instead, a 220 m length of 12 mm Dyneema rope was used, with midwater buoyancy set at 100 m above the seabed to create a false bottom, and improved chain weight dampening at the seabed to create elasticity.

## Data Records

The data records are organised by deployment and labelled Southern Ocean Wave Buoy Data: Deployment 1, Deployment 2 and Deployment 3. Complete data records have been archived with Marine Data Archives (MDA)^10^ in the same Triaxys file format as received from the buoy, which includes the 2D wave spectra and Fourier coefficients. The processed wave spectral estimates have been archived with the Australian Ocean Data Network^11^ (AODN) in the standard Triaxys parameter convention. A list of the Triaxys parameters is provided in Table 1, including a brief description of each. For both data archives, the time stamp is UTC and magnetic correction has not been applied to directions in the data.

**Table 1:** Description of the named Triaxys wave spectral estimates.

Parameter name	Units	Description
**Zero crossings**		Number of waves detected by zero-crossing analysis of the wave elevation record.
**Ave. Ht**.	m	Average zero down-crossing wave height.
**Ave. Per**.	s	Average zero down-crossing wave period.
**Max Ht**.	m	Maximum zero down-crossing wave height (trough to peak).
**Sig. Wave**	m	Zero down-crossing significant wave height, Hs, where Hs is the average height of the highest third of the waves.
**Sig. Per**	s	Average period of the significant zero down-crossing waves.
**Peak Per. (Tp)**	s	Peak wave period Tp = 1.0/fp where fp is the frequency at which the wave spectrum S(f) has its maximum value.
**Peak Per. (READ)**	s	Peak wave period as computed by the Read method.
**HM0**	m	Significant wave height as estimated from spectral moment mo. Hmo = 4.0 * SQRT(m0) where m0 is the integral of S(f)*df from f = F1 to F2 Hz.
**Mean Theta**	degrees	Overall mean wave direction in degrees obtained by averaging the mean wave angle θ over all frequencies with weighting function S(f). θ is calculated by the KVH method.
**Sigma Theta**	degrees	Overall directional spreading width in degrees obtained by averaging the spreading width sigma theta, σθ, over all frequencies with weighting function S(f). σθ is calculated by the KVH method.

Researchers seeking standard wave spectral estimates are encouraged to access files via the AODN. However, if detailed spectra or an alternative processing of spectra is required, then users should access the files from the MDA. For processing of spectra, the open-source code *Wavespectra* is recommended as this will directly read the native Triaxys files. *Wavespectra* can be found at GitHub (https://github.com/wavespectra/wavespectra) and Zenodo^12^.

**Fig. 2:**
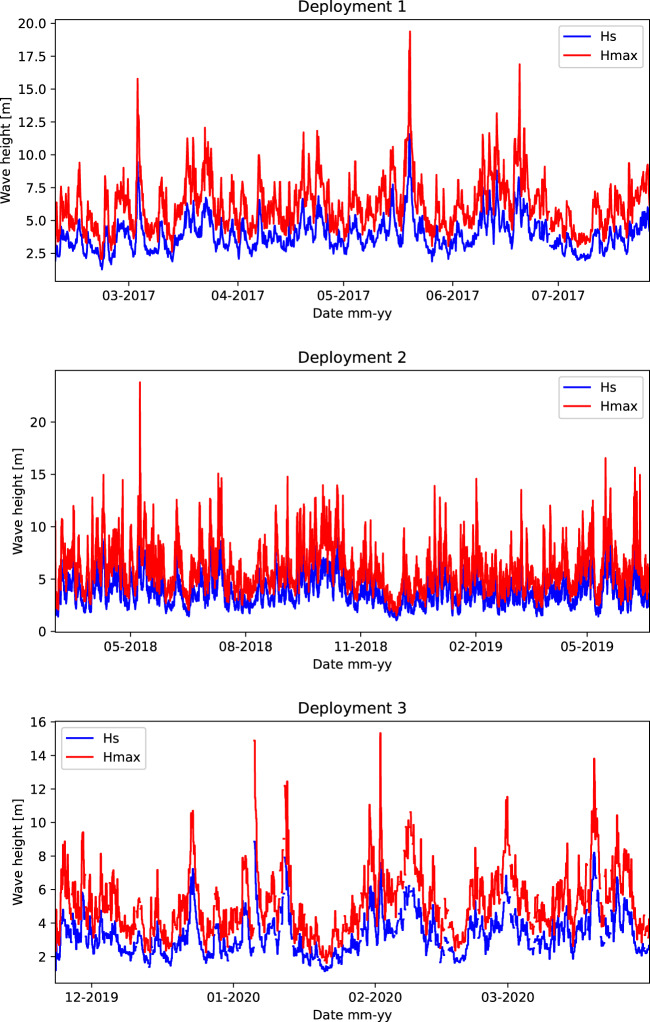
Time series plots showing the measured significant and maximum wave heights during the three deployments. The upper plot presents the observations from 08/02/2017 to 27/07/2017, middle plot is from 02/03/2018 to 19/02/2019, and the lower plot is from 25/11/2019 to 25/04/2020. The largest observed maximum wave height during the observation program to date is 23.8 m.

**Fig. 3:**
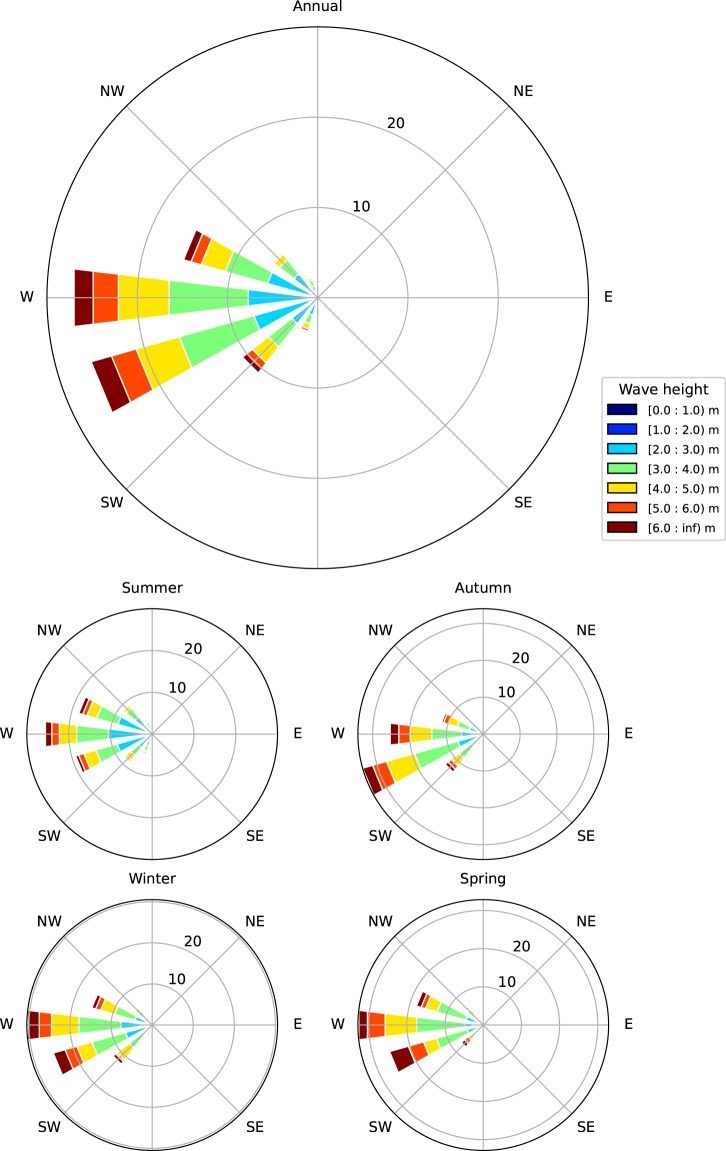
Annual and austral seasonal wave roses. Observations from the three deployments are presented as wave rose plots for the total period (upper plot) and for each month. Throughout the year, the measured wave directions are dominated by the West and South-westerly octants.

Time series plots of the measured significant (HMO) and maximum (HMAX) wave height are provided in Fig. 2 for the 784 days of data from the three deployments. Statistics from the data set are presented in Tables 2–4, providing a summary of the monthly wave height values, the annual joint probability distribution of significant wave height and wave direction, and significant wave height and peak wave period. Annual and monthly roses are presented in Fig. 3. Note that magnetic correction for direction has been applied in these tables and figures, using the methodology provided in Guedes *et al*.^12^.

**Table 2:** Observed monthly significant wave height statistics (m).

	*N* obs	max	mean	p1	p10	p50	p90	p95	p99
**Jan**	472	8.82	3.48	1.33	1.90	3.22	5.37	6.18	7.70
**Feb**	620	8.32	3.53	1.64	2.19	3.40	4.88	5.86	7.40
**Mar**	744	9.44	3.70	1.60	2.44	3.50	5.37	6.08	7.45
**Apr**	673	9.90	3.76	1.97	2.49	3.50	5.40	6.16	8.01
**May**	722	14.94	4.24	1.84	2.44	3.96	6.45	7.31	9.78
**Jun**	630	9.71	4.02	1.63	2.39	3.76	6.00	6.87	8.20
**Jul**	430	8.63	3.76	2.02	2.32	3.48	5.63	6.75	8.12
**Aug**	248	8.37	3.45	1.86	2.38	3.14	4.96	5.56	6.58
**Sep**	240	8.14	4.54	2.36	2.92	4.29	6.58	7.27	7.98
**Oct**	241	8.88	4.48	1.99	2.46	4.27	6.60	6.93	8.56
**Nov**	276	6.85	3.09	1.13	1.54	3.17	4.46	4.96	6.33
**Dec**	485	7.84	2.97	1.65	1.94	2.74	4.15	4.84	7.09
**All**	5781	14.94	3.75	1.54	2.26	3.48	5.69	6.44	8.10

**Table 3:** Joint probability distribution (%) of observed significant wave height and wave direction.

Hs (m)	Wave direction (degT)								Sum	Exceed%
	337.5–22.5	22.5–67.5	67.5–112.5	112.5–157.5	157.5–202.5	202.5–247.5	247.5–292.5	292.5–337.5		
**0.5**–**1**	—	—	—	—	—	—	—	—	—	100.0
**1**–**1.5**	0.09	0.06	0.09	0.07	0.32	0.28	0.09	0.24	1.24	100.0
**1.5**–**2**	0.38	0.26	0.22	0.11	0.56	1.26	1.94	1.11	5.84	98.78
**2**–**2.5**	0.88	0.41	0.16	0.18	0.5	2.98	5.99	2.61	13.71	93.06
**2.5**–**3**	0.40	0.37	0.33	0.18	0.87	3.93	7.06	2.57	15.71	79.52
**3**–**3.5**	0.26	0.44	0.38	0.17	0.72	4.38	7.98	2.31	16.64	63.83
**3.5**–**4**	0.27	0.07	0.10	0.04	0.37	3.93	7.8	2.15	14.73	47.27
**4**–**4.5**	0.09	0.04	0.13	0.02	0.32	2.65	5.92	1.13	10.30	32.39
**4.5**–**5**	0.09	0.01	0.05	0.04	0.12	2.05	4.23	0.82	7.41	22.08
**5**–**5.5**	0.02	—	—	0.01	0.12	1.16	2.67	0.32	4.30	14.58
**5.5**–**6**	—	—	0.01	—	0.15	1.02	2.45	0.28	3.91	10.20
**6**–**6.5**	0.02	—	—	—	0.05	0.63	1.38	0.13	2.21	6.38
**6.5**–**7**	—	—	—	—	0.05	0.35	1.16	0.07	1.63	4.07
**7**–**7.5**	—	—	—	—	0.01	0.27	0.52	0.02	0.82	2.42
**7.5**–**8**	—	—	—	—	0.02	0.22	0.40	0.07	0.71	1.57
**8**–**8.5**	—	—	—	—	0.02	0.11	0.28	0.05	0.46	0.84
**8.5**–**9**	—	—	—	—	0.02	0.06	0.06	0.05	0.19	0.38
**9**–**9.5**	—	—	—	—	—	0.01	0.06	—	0.07	0.18
**9.5**–**10**	—	—	—	—	—	0.01	0.01	—	0.02	0.11
**10**–**10.5**	—	—	—	—	0.01	—	—	0.01	0.02	0.09
**10.5**–**11**	—	—	—	—	—	—	—	—	—	0.06
**11**–**11.5**	—	—	—	—	0.01	—	—	—	0.01	0.06
**11.5**–**12**	—	—	—	—	—	0.01	0.02	—	0.03	0.05
**12**–**12.5**	—	—	—	—	—	—	—	—	—	0.01
**12.5**–**13**	—	—	—	—	—	—	—	—	—	0.01
**13**–**13.5**	—	—	—	—	—	—	—	—	—	0.01
**13.5**–**14**	—	—	—	—	—	—	—	—	—	0.01
**14**–**14.5**	—	—	—	—	—	—	—	—	—	0.01
**14.5**–**15**	—	—	—	—	—	—	—	0.01	0.01	0.01
**Total**	2.50	1.66	1.47	0.82	4.24	25.31	50.02	13.95	100.0	—
**Exceed%**	1.56	99.07	97.41	95.95	95.12	90.89	65.58	15.54	—	—

**Table 4:** Joint probability distribution of observed significant wave height and peak wave period.

Hs (m)	Peak wave period (s)									Sum	Exceed%
	4–6	6–8	8–10	10–12	12–14	14–16	16–18	18–20	20–22		
**0.5–1**	—	—	—	—	—	—	—	—	—	—	100.0
**1–1.5**	—	0.06	0.45	0.46	0.24	0.01	—	—	—	1.22	100.0
**1.5–2**	0.10	0.77	2.03	1.34	1.28	0.27	0.05	—	—	5.84	98.78
**2–2.5**	0.17	1.63	4.48	3.59	2.48	1.00	0.3	0.06	—	13.71	93.06
**2.5–3**	—	1.16	5.32	4.10	3.43	1.40	0.29	0.01	—	15.71	79.52
**3–3.5**	—	0.61	4.88	4.99	3.78	2.10	0.27	—	—	16.63	63.83
**3.5–4**	—	0.11	3.84	4.10	3.92	2.42	0.32	0.01	—	14.72	47.27
**4–4.5**	—	0.01	2.45	3.42	2.94	1.37	0.11	—	—	10.30	32.39
**4.5–5**	—	0.04	1.18	2.45	2.56	1.02	0.13	0.01	—	7.39	22.08
**5–5.5**	—	—	0.51	1.06	1.71	0.85	0.17	—	—	4.30	14.58
**5.5–6**	—	—	0.09	1.06	1.52	1.12	0.12	—	—	3.91	10.20
**6–6.5**	—	—	0.02	0.37	1.04	0.67	0.12	—	—	2.22	6.38
**6.5–7**	—	—	—	0.17	0.65	0.70	0.12	—	—	1.64	4.07
**7–7.5**	—	—	—	0.02	0.21	0.50	0.06	0.04	—	0.83	2.42
**7.5–8**	—	—	—	0.01	0.35	0.29	0.06	—	—	0.71	1.57
**8–8.5**	—	—	—	0.02	0.11	0.27	0.06	—	—	0.46	0.84
**8.5–9**	—	—	—	—	0.02	0.10	0.07	—	—	0.19	0.38
**9–9.5**	—	—	—	—	—	0.05	0.02	—	—	0.07	0.18
**9.5–10**	—	—	—	—	—	0.01	0.01	—	—	0.02	0.11
**10–10.5**	—	—	—	—	0.01	—	0.01	—	—	0.02	0.09
**10.5–11**	—	—	—	—	—	—	—	—	—	—	0.06
**11–11.5**	—	—	—	—	—	—	0.01	—	—	0.01	0.06
**11.5–12**	—	—	—	—	—	0.01	—	0.02	—	0.03	0.05
**12–12.5**	—	—	—	—	—	—	—	—	—	—	0.01
**12.5–13**	—	—	—	—	—	—	—	—	—	—	0.01
**13–13.5**	—	—	—	—	—	—	—	—	—	—	0.01
**13.5–14**	—	—	—	—	—	—	—	—	—	—	0.01
**14–14.5**	—	—	—	—	—	—	—	—	—	—	0.01
**14.5–15**	—	—	—	—	—	—	0.01	—	—	0.01	0.01
**Total**	0.27	4.39	25.25	27.16	26.25	14.16	2.31	0.15	—	100.0	—
**Exceed%**	100.0	99.73	96.1	82.34	42.92	16.66	2.50	0.16	0.07	—	—

## Technical Validation

The buoy data described in the previous sections were compared against concurrent satellite altimeter passes to verify the quality of the observations. The colocation was calculated as the average of all altimeter measurements inside a circle of 0.5-degree radius within a 1-hour window centred on the timestamp of the buoy measurement. Note this validation must consider the expected uncertainties in the reference altimetry data, as well as differences caused by the different spatio-temporal characteristics of buoy and altimeter measurements^13^.

**Fig. 4:**
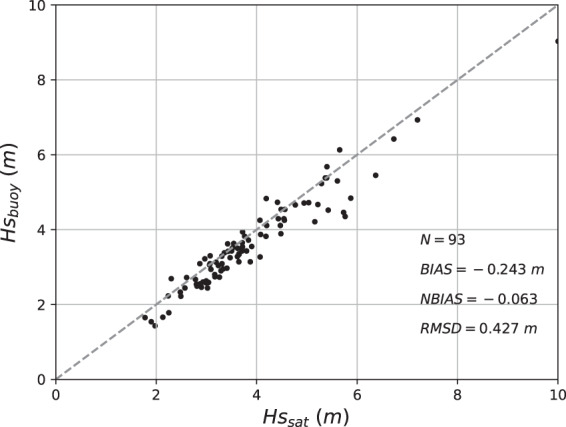
Scatter plot of wave buoy significant wave heights vs the satellite altimetry derived values during the three deployment periods. Co-located data within 0.5-degree radius and 1-hour were sourced from SARAL, Jason2, Jason3, Cryosat2 and Sentinel3A.

Significant wave height measured by the moored wave buoy compares well against satellite observations, as shown in Fig. 4. The satellite datasets used were SARAL, Jason2 and Jason3 (from AVISO), Cryosat2 (from Globwave) and Sentinel3A (from Copernicus). The overall RMSD was 0.427 m and the bias was 0.243 m, corresponding to a normalised bias of 0.063. Note the satellite footprint included the nearby island, which can affect wave height estimates from altimeters.

The wave rose (Fig. 3) shows that the predominant direction of approach for waves is the west and southwest sectors. This quadrant is not affected by the presence of the island to the north of the buoy. We note that wave energy arriving from the north sector will likely be attenuated by the island to some degree, however this directional sector is not common nor particularly energetic compared with the west and southwest sectors. Our directional observations are in qualitative agreement with Rapizo *et al*.^5^, who noted a predominance of southwest sector waves and showed that the north through east sector waves were very infrequent and typically of low energy. The mean significant wave height from the SOFS programme was 4.09 m, while the mean value observed here was 3.75 m. Both sites are dominated by peak spectral wave period in the range 10–14 s.

## Usage Notes

The standard wave spectral estimates may be downloaded from the AODN, while the raw files including 2D spectra are available from the MDA. Note that this is an ongoing observation programme and updates will be made to both repositories at annual intervals.

## Data Availability

No custom code was used to generate or process the data described in this manuscript.
